# Who Benefits From a Defibrillator—Balancing the Risk of Sudden Versus Non-sudden Death

**DOI:** 10.1007/s11897-018-0416-6

**Published:** 2018-11-10

**Authors:** Simon A. S. Beggs, Roy S. Gardner, John J. V. McMurray

**Affiliations:** 1 British Heart Foundation Cardiovascular Research Centre, University of Glasgow, Glasgow, Scotland; 2 Golden Jubilee National Hospital, Clydebank, Scotland

**Keywords:** Defibrillator, ICD, Sudden death, Competing risk

## Abstract

**Purpose of Review:**

Treatment with a defibrillator can reduce the risk of sudden death by terminating ventricular arrhythmias. The identification of patient groups in whom this function reduces overall mortality is challenging. In this review, we summarise the evidence for who benefits from a defibrillator.

**Recent Findings:**

Recent evidence suggests that contemporary pharmacologic and non-defibrillator device therapies are altering the potential risks and benefits of a defibrillator.

**Summary:**

Who benefits from a defibrillator is determined by both the risk of sudden death and the competing risk of other, non-sudden causes of death. The balance of these risks is changing, which calls into question whether historic evidence for the use of defibrillators remains robust in the modern era.

## Introduction

In 1980, Mirowski et al. reported the first three patients treated with an automatic implantable defibrillator (AID) [1]. This early AID was a ‘shock box’, delivering only high-voltage defibrillation therapy. Later devices evolved to include a pacing function and are now usually described as implantable cardioverter defibrillators (ICDs). The randomised controlled trials (RCTs) that followed examined the efficacy of ICD therapy in populations targeted for their high incidence of sudden cardiac death (SCD). Specifically, these were patients with prior cardiac arrest, ventricular arrhythmias (VA) or myocardial infarction (MI), or the presence of coronary artery disease (CAD), left ventricular systolic dysfunction (LVSD), heart failure or some combination of these problems. Incrementally, these RCTs established our current understanding of which patients are more, or less, likely to benefit from an ICD. The importance of several major themes that progressively emerged from these trials must be recognised if a thorough examination of this evidence is undertaken. These include the role of competing risks of death, appropriate trial design—including length of follow-up and the selection of a suitable primary endpoint—the technical evolution of the defibrillator, and incremental advances in prognostic pharmacologic and additional device therapy.

## Who Benefits From a Secondary Prevention Defibrillator?

The benefit of an ICD as a secondary prevention treatment was examined in three major prospective, multi-centre RCTs. Summary characteristics of these studies are presented in Table 1. The first published and largest study was the Antiarrhythmics Versus Implantable Defibrillators (AVID) trial [2**•**]. Patient eligibility in AVID required one of the following: (1) resuscitation from ventricular fibrillation (VF), (2) syncope with sustained ventricular tachycardia (VT) or (3) sustained VT with significant associated symptoms and left ventricular ejection fraction (LVEF) ≤ 40%. AVID enrolled 1016 patients, randomising them to treatment with an ICD or antiarrhythmic drugs (AADs), primarily amiodarone. Over a mean follow-up of 18 months, there were 80 and 122 deaths from any cause in the ICD and AAD arms, respectively (hazard ratio [HR] 0.62, 95% confidence intervals [CI] 0.47–0.81, *p* < 0.02). This reduction in all-cause mortality (ACM) was driven by a reduction in SCD (HR 0.43, 95%CI 0.27–0.66). The Cardiac Arrest Study Hamburg (CASH) trial enrolled 346^1^ patients resuscitated from cardiac arrest due to VT/VF, randomising them to receive an ICD or one of three AADs: amiodarone, metoprolol or propafenone [3]. Finally, the Canadian Implantable Defibrillator Study (CIDS) recruited 659 patients who had experienced VT/VF associated with one of five eligible clinical presentations (see Table 1) [4]. Patients were randomised to an ICD or amiodarone in a 1:1 ratio. Neither CASH nor CIDS demonstrated a significant reduction in ACM with ICD therapy, although treatment with a defibrillator was associated with a lower risk of SCD in CASH (HR 0.32, 95%CI 0.15–0.69).

**Table 1:** Outline characteristics of randomised controlled trials utilising defibrilators as primary or secondary prevention therapy

	Secondary prevention trials			Primary prevention trials–ischaemic aetiology						Primary prevention trials–nonischaemic or mixed aetiology					
Trial name	AVID [2•]	CASH [3]	CIDS [4]	MADIT [5]	CABG-Patch [6]	MUSTT [7]	MADIT II [8•]	DINAMIT [9•]	IRIS [10]	CAT [11]	AMIOVIRT [12]	DEFINITE [13]	COMPANION [14]	SCD-HeFT [15•]	DANISH [16•]
Year of publication	1997	2000	2000	1996	1997	1999	2002	2004	2009	2002	2003	2004	2004	2005	2016
Years of enrollment	1993–1997	1987–1996	1990–1997	1990 - NR	1990 - NR	1990–1997	1997–2001	1998–2002	1999–2007	1991–97	1996–2000	1998–2002	2000–2002	1997–2001	2008–2014
Inclusion criteria	i) Resuscitated VF; OR ii) susVT + syncope; OR iii) symptomatic susVT + LVEF ≤ 40%	CA due to VA	i) documented VF; OR ii) CA requiring defibrillation iii) susVT + syncope; OR iv) susVT + angina or presyncope if LVEF ≤ 35%; OR v) unmonitored syncope + subsequent spontaneous VT or PVS-induced susVT	Previous MI (> 3 weeks) + asymptomatic nsVT, EPS+ despite procainamide suppression	Planned CABG + SAE positive	CAD + PVS-induced susVT	Previous MI (> 1 month)	Recent MI (6–40 days) + CAF+	Recent MI (5–31 days) and: i) LVEF ≤ 40% and HR ≥ 90 on ECG; OR ii) nsVT on Holter	NICM ≤9 months + NYHA II or III	NICM + NYHA I to III + asymptomatic nsVT	NICM + prev HF + nsVT or ≥ 10 PVCs per hour	QRS interval ≥ 120 ms + PR interval ≥ 150 ms + sinus rhythm + recent HFH	As per LVEF and NYHA criteria below	NICM + NT-proBNP > 200 pg/ml
LVEF cut-off	N/A	N/A	N/A	≤ 35%	≤ 35%	≤ 40%	≤ 30%	≤ 35%	As above	≤ 30%	≤ 35%	≤ 35%	≤ 35%	≤ 35%	≤ 35%
NYHA criteria	N/A	N/A	N/A	I to III	I to IV	I to III	I to III	I to III	I to III	II to III	I to III	I to III	III or IV	II or III	II or III (or IV if CRT planned)
No. randomised	1016	288**	659	196	900	704	1232	674	898	104	103	458	1520	2521	1116
No. (%) with NICM	193 (19.0)	220	63 (9.6)	0	0	0	0	0	0	104 (100)	103 (100)	458 (100)	678 (45)	1211 (48)	1116 (100)
Follow-up, mean (SD), month	18.2 (12.2)	57 (34)	35	27	32 (16)	39	20	30 (13)	37	66 (26.4)	24 (14.4)	29 (14)	Range 14.8–16.0	45.5	67.6 (NR)
Demographics															
Age, mean (SD), year	65 (10.5)	56	64 (9.6)	63	64 (9)	67	64 (10)	62 (11)	63	52 (11)	59 (11.5)	58	67	60	64*
Male, % (No.)	80 (808)	80 (230)	84.5 (557)	92 (172)	16 (759)	90	84 (1040)	76 (514)	77 (689)	80 (83)	70 (72)	71 (326)	67 (1025)	77 (1933)	72 (809)
NYHA class III/IV, % (No.)	9.5 (97)	16 (47)	10.8 (71)	NR	NR	24	29 (355)	13 (89)	12 (107)	34.6 (36)	20 (20)	21 (96)	100 (1520)	30 (760)	47 (519)
Duration of CHF, mean	NR	NR	NR	NR	n/a	NR	NR	NR	332	3 months	3.2 years	2.8 years	3.6 years	24.5 months	19 (NR)*
LVEF, mean (SD), %	31 (13)	46 (18)	34 (14)	26 (7)	27 (6)	30	23 (5)	28 (5)	342	24 (7)	23 (9)	21 (14)	21	25	25 (NR)*
Medications at baseline, % (No.)									674						
ACE inhibitor/ARB	68.5 (680)	45 (125)	NR	58 (107)	53 (473)	75 (525)	70 (858)	95 (638)	82 (734)	96.2 (100)	85 (88)	96.7 (443)	89 (1359)	96 (2432)	97 (1077)
BB	29.4 (292)	33 (96)	27.4 (181)	17 (31)	20 (183)	40 (282)	70 (862)	87 (585)	87 (782)	3.8 (4)	51.5 (53)	84.9 (389)	68 (1027)	69 (1738)	92 (1026)
MRA	NR	NR	NR	NR	NR	NR	NR	NR	NR	NR	19.4 (20)	NR	54 (824)	20 (507)	58 (646)
CRT	0	0	0	0	0	0	0	0	0	0	0	4.8 (11) in ICD group; NR in controls	80 (1212) intreatment groups	NR	58 (645)
Design	ICD vs antiarrhythmic	ICD vs amio. vs BB	ICD vs amio.	ICD vs CMT	ICD vs CMT	EPS+ guided assignment to ICD or AAD, or standard care if negative EPS	ICD vs CMT (3:2)	ICD vs CMT	ICD vs CMT	ICD vs CMT	ICD vs amio.	ICD vs CMT	CRT-D vs CRT-P vs CMT	ICD vs amio. vs placebo	ICD vs CMT
Primary endpoint	ACM	ACM	ACM	ACM	ACM	CA or arrhythmic death	ACM	ACM	ACM	ACM	ACM	ACM	ACM/ACH	ACM	ACM
Defibrillator															
Transvenous/epicardial, %	93/5	44/56	84/10	53 / 47	0/100	NR	100/0	100/0	100/0	100/0	100/0	100/0	100/0	100/0	100/0
Internal validity															
Follow-up, %	100	100	100	99	100	NR	NR	99%	99.9%	100	100	100	> 95%	100	100
Crossovers to ICD, % (No.)	18.9 (96)†	5.8 (11)	15.7 (52)	11 (11)	4 (18)	12 (n/a)	4.5 (22)	0	8.6 (39/453)	NR	15.4 (8)	10 (23)	26 (80)††	NR	4.8 (27)
Crossovers to control % (No.)	25.7 (130)	6.1 (6)	28.1 (92)	5 (5)	12 (52)	n/a	6 (44)	6 (20)	10 (45/445)	NR	21.6 (11)	1.7 (4)	NR	NR	7.9 (44)

*AAD*, antiarrhythmic drug; *ACE*, angiotensin-converting enzyme; *ACH*, all-cause hospitalisation; *ACM*, all-cause mortality; *Amio*., amiodarone; *ARB*, angiotensin-receptor blocker; *BB*, beta-blocker; *CA*, cardiac arrest; *CAF+*, positive cardiac autonomic function test; *CHF*, congestive heart failure; *CMT*, conventional medical therapy; *CRT-D*, cardiac resynchronisation therapy defibrillator; *CRT-P*, cardiac resynchronisation therapy pacemaker; *EPS+*, nonsuppressible ventricular tachyarrhythmia on electrophysiologic study; *HFH*, hospitalisation for heart failure; *ICD*, implantable cardioverter defibrillator; *LVEF*, left ventricular ejection fraction; *MRA*, mineralocorticoid receptor antagonist; *NICM*, non-ischaemic cardiomyopathy; *NR*, not reported; *NYHA*, New York Heart Association; *SAE*+, abnormalities on a signal-averaged electrocardiogram; *PVS*, programmed ventricular stimulation; *AVID*, Antiarrhythmics Versus Implantable Defibrillators; *CASH*, Cardiac Arrest Study Hamburg Trial; *CIDS*, Canadian Implantable Defibrillator Study; *MADIT*, Multicentre Automatic Defibrillator Implantation Trial; *MADIT II*, Second Multicentre Automatic Defibrillator Implantation Trial; *DINAMIT*, Defibrillator in Acute Myocardial Infarction Trial; *IRIS*, Immediate Risk Stratification Improves Survival Trial; *CABG-Patch*, Coronary Artery Bypass Graft Patch Trial; *MUSTT*, Multicentre Unsustained Tachycardia Trial; *AMIOVIRT*, Amiodarone Versus Implantable Cardioverter-Defibrillator; *CAT*, Cardiomyopathy Trial; *DEFINITE*, Defibrillators in Non-Ischaemic Cardiomyopathy Treatment Evaluation Trial; *SCD-HeFT*, The Sudden Cardiac Death in Heart Failure Trial; *COMPANION*, The Comparison of Medical Therapy, Pacing, and Defibrillation in Heart Failure; *DANISH*, Danish Study to Assess the Efficacy of ICDs in Patients with Non-ischemic Systolic Heart Failure on Mortality

*Calculated as mean of stated medians for treatment and control groups

^†^For AVID, crossover rates are reported at 2 years

^††^For COMPANION, rate of withdrawal from medical therapy group is reported as crossover rate

**Randomised to ICD, amiodarone, or metoprolol (not propafenone)

***Non-defibrillator group

One thousand eight hundred sixty-six participants from AVID, CIDS and CASH were subsequently combined in a patient-level meta-analysis evaluating the effects of randomisation to an ICD versus amiodarone or sotalol (thus excluding the 97 patients allocated to metoprolol in CASH) [17•]. Treatment with an ICD conferred a lower risk of ACM (HR 0.73, 95%CI 0.60–0.87, *p* < 0.001), with this effect being driven by a reduction in SCD (HR 0.49, 95%CI 0.36–0.67, *p* < 0·001). There was no significant difference in non-sudden mortality between the groups. The treatment effect of an ICD was consistent across the three trials (*p* value for interaction = 0.306), suggesting that CIDS and CASH provide supportive evidence for, rather than militating against, the survival benefit seen in AVID. Another meta-analysis of the secondary prevention trials which included an additional small Dutch study estimated a relative risk of ACM of 0.75, (95%CI 0.64–0.87, *p* = 0·0002) [18].

**Fig. 1:**
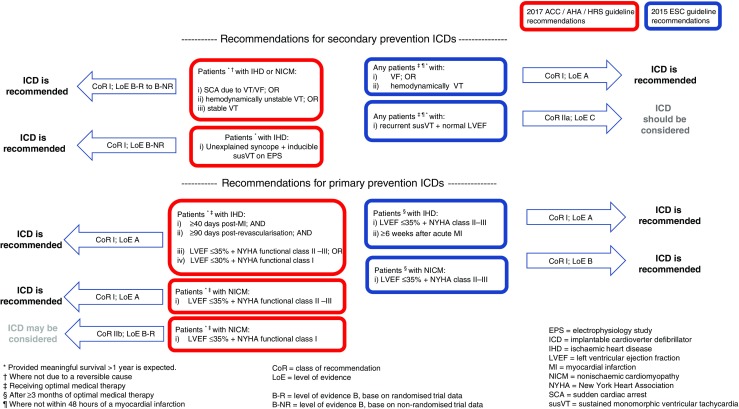
Selected international guideline recommendations for implantable cardioverter defibrillators

These trials form the basis for international guideline recommendations [19, 20] for secondary prevention ICDs, including those from the European Society of Cardiology (ESC) and a combined guideline from the American College of Cardiology (ACC), American Heart Association (AHA) and the Heart Rhythm Society (HRS). Selected recommendations from these guidelines are summarised and contrasted in Fig. 1.

As to precisely who benefits from a secondary prevention ICD, there is little more to add. Exploration of subgroups in the first meta-analysis described above found no interaction between treatment effect and index VA, prior MI, non-ischemic aetiology, coronary artery disease, New York Heart Association (NYHA) functional class or coronary artery bypass at baseline. However, two significant subgroup interactions were identified. First, patients treated with an ICD during the ‘epicardial era’—defined as ending on 1st July 1991 – derived less benefit from a defibrillator than those randomised after this date, who largely received transvenous systems (*P* for interaction = 0.029). Although this effect is likely confounded by other advances in management pre- and post-1991, the proposition that transvenous ICDs should be superior to epicardial systems—which require a thoracotomy—is patently plausible. Today, epicardial ICDs are seldom implanted. Second, patients with LVEF ≤ 35% derived significantly more benefit from a defibrillator than those with LVEF > 35% (*P* for interaction = 0.011), in whom there was a non-significant trend towards harm (HR 1.2, 95%CI 0.81–1.76). The CASH trial—in which more than half of patients received an epicardial system—enrolled a population with a significantly higher mean LVEF than AVID or CIDS (46% vs 31% and 34% respectively). It is therefore possible that the lower efficacy of an ICD detected in the LVEF > 35% group was actually due to more frequent epicardial ICD use in this population. In any case, subgroup analyses must obviously be treated with caution: consequently, international guideline recommendations for secondary prevention ICDs do not differentiate according to LVEF, and further evidence from prospective studies would be required before this would change.

A final point of interest is that, in this meta-analysis, the incremental separation over time of the Kaplan-Meier curves for arrhythmic death contrasts with the lack of progressive divergence between the curves for ACM, which initially separates before starting to converge after 4 years. Although this suggested that the benefit of an ICD might wane over a longer period, further insight is curtailed by the relatively short follow-up in AVID and CIDS.

## Who Benefits From a Primary Prevention Defibrillator?

### Acute MI: Late Implantation of an ICD

The quest to identify further patient groups which might benefit from an ICD led next to primary prevention trials in patients with a history of acute myocardial infarction (AMI). At the time—approximately three decades ago—4 to 5-year mortality following hospital discharge after AMI was ≥ 20% amongst patients with LVSD [21–23], with SCD accounting for roughly one third of late mortality [21, 24]. Seminal amongst these new trials was the Second Multicentre Automatic Defibrillator Implantation Trial (MADIT II) [8•]. Although three prior RCTs [5–7] had examined the benefit of an ICD in patients with CAD and/or MI, all had required the presence of VT and/or an abnormal signal-averaged electrocardiogram (SAECG) (see Table 1). Inclusion criteria for the original Multicentre Automatic Defibrillator Implantation Trial (MADIT I), for example, had included not merely LVSD and a prior AMI, but also asymptomatic non-sustained VT and inducible, non-suppressible VT on an electrophysiology (EP) study. These predecessor trials were thus less pragmatic and less broadly applicable than MADIT II, in which eligible patients had experienced an AMI 1 month or more prior to enrolment (although in three-quarters of patients the gap was 18 months prior or longer, as shall be discussed later), had an LVEF ≤ 30%, and had not undergone coronary revascularisation within the preceding 3 months. Participants were assigned in a 3:2 ratio to receive either an ICD (*n* = 742) or conventional medical therapy (CMT) (*n* = 490), amongst whom only 10% were prescribed amiodarone.

During a mean follow-up of 20 months, the rates of ACM were 14.2% and 19.8% in the ICD and CMT groups respectively, with an ICD conferring a 31% relative reduction in the risk of death (HR 0.69, 95%CI 0.51–0.93, *p* = 0.016). The effect of ICD therapy on survival did not vary according to any of the subgroups analysed, including those defined by age, sex, LVEF, NYHA class, QRS duration, diabetes, left bundle-branch block or atrial fibrillation. The survival benefit was entirely due to a reduction in SCD, consistent with the plausible benefit of a defibrillator (HR 0.33, 95%CI 0.20–0.53, *p* < 0.0001) [25]. Notably, although the cumulative hazard curves reflecting SCD separated early, those for ACM diverged only after ~ 9 months. This effect appears to have been driven by a trend towards increased non-sudden cardiac death within the ICD group, especially during the first year, although this did not reach statistical significance. There was also a trend to new or worsening HF in the ICD group.

### Acute MI: Early Implantation of an ICD

Patients within 1 month of an AMI were excluded from MADIT II. Two subsequent RCTs—the Defibrillator in Acute Myocardial Infarction Trial (DINAMIT) and Immediate Risk Stratification Improves Survival (IRIS) trial—thus sought to examine the effect of an ICD in patients following a recent AMI (6–40 days and 5–31 days post-MI respectively) [9•, 10]. Outline characteristics of both trials are presented in Table 1. Eligibility criteria for DINAMIT included LVEF ≤ 35% and impaired cardiac autonomic function, with patients randomised to medical therapy with (*n* = 332) or without (*n* = 342) a defibrillator. After an average follow-up of 30 months, there was a 58% lower risk of SCD in the ICD group when compared to the control group (HR 0.42, 95%CI 0.22–0.83, *p* = 0.009), with 12 and 29 sudden deaths respectively in these groups. An ICD did not confer an overall mortality benefit however, due to a significant increase in non-sudden cardiac deaths in the ICD group (HR 1.75, 95%CI 1.11–2.76, *p* = 0.02). IRIS required eligible patients to have LVEF ≤ 40% and a heart rate ≥ 90 beats per minute or non-sustained VT on Holter monitor, randomising them to medical therapy with (*n* = 445) or without (*n* = 453) a defibrillator. During an average follow-up of 37 months, there was a lower risk of sudden death with an ICD (HR 0.55, 95%CI 0.31–1.00, *p* = 0.049) that, as in DINAMIT, was offset by an increase in non-sudden cardiac death amongst ICD recipients (HR 1.92, 95%CI 1.29–2.84, *p* = 0.001). Resultantly, there was no difference in ACM between the two groups.

This increase in non-sudden death associated with an ICD in DINAMIT and IRIS is partially explained by the competing risks posed by multiple potential causes of death. In the ICD groups, the reduction in SCD associated with a defibrillator led to a larger pool of survivors at risk of non-sudden death when compared to the control groups. That is, treatment with an ICD may have merely switched the mode of death from arrhythmic to non-arrhythmic (presumably deaths due to ischaemia or heart failure since the difference in non-sudden events was driven by cardiac causes). This is why ACM—rather than SCD—is the more appropriate primary endpoint for a trial examining the benefit of a defibrillator.^2^ Of course, this principle is true of other ICD trials, yet in several increases in non-sudden death were not sufficiently great to offset the concomitant reductions in SCD afforded by an ICD. For example, non-sudden cardiac death increased in the ICD arm of MADIT II (4.3% of deaths in the control group, compared to 5.8% of deaths in the ICD group) without nullifying the overall survival benefit of an ICD.

Closer inspection of these trials is instructive. In both DINAMIT and IRIS, clinical events were classified by adjudication committees blinded to treatment assignment; there were few significant differences between the ICD and control group characteristics, and neither trial experienced high cross-over rates between treatments. Artefact was therefore unlikely to have substantially contributed to the results. The first major difference in inclusion criteria between DINAMIT/IRIS and other primary prevention trials carried out in patients with ischemic heart disease (IHD) was the use of autonomic function and heart rate criteria in the former (depressed heart-rate variability or elevated 24-h heart rate in DINAMIT and elevated heart rate or non-sustained VT in IRIS). Subsequent research suggests that these phenomena are associated with an increased risk of ACM rather than specifically arrhythmic SCD [26, 27]. Thus, whereas the investigators had intended these criteria to select participants at particularly high risk of arrhythmic death, they may have inadvertently enriched the study populations with patients also at high risk of non-sudden cardiac death, thus increasing the probability that patients ‘saved’ from arrhythmic death by an ICD would nevertheless die from an alternative cardiac cause soon after.

The second major difference in inclusion criteria between DINAMIT/IRIS and other primary prevention trials performed in IHD populations was the temporal proximity to index AMI. This temporal difference is more substantial than might be assumed a priori from inclusion criteria. For example, although MADIT II ostensibly recruited patients who were at least 1 month remote from an AMI, in reality, this period was 81 months on average, and greater than 6 months in nearly 90% of patients [28]. The subpopulation of DINAMIT and IRIS patients who died during the early post-AMI period were thus not meaningfully represented in MADIT II. This survivorship bias is important because this cohort is likely to have had a particularly vulnerable cardiac substrate, susceptible not merely to ventricular arrhythmias but to other causes of cardiac death. By comparison, it may be presumed that patients in other primary prevention RCTs had more compensated, remodelled hearts that were susceptible to scar-related VAs but at relatively lower competing risk of imminent reinfarction or heart failure. Substantiating this, a subgroup analysis of MADIT-II found that although mortality risk increased as a function of time from MI, randomisation to an ICD only conferred a survival benefit in those patients ≥ 18 months remote from their qualifying MI (HR 0.55, 95%CI 0.39–0.78, *p* = 0.001) versus those with relatively recent MI (< 18 months; HR 0.97, *p* = 0.92), with this benefit remaining substantial for ≥ 15 years post-infarct [28].

A further, more troubling possibility is that ICDs might increase non-arrhythmic mortality not merely indirectly, by ‘switching’ the mode of death, but directly, via intrinsically deleterious mechanisms. This relates less to peri-procedural deaths—of which there was only one in IRIS and none in DINAMIT—but rather the consequences to the myocardium of high voltage defibrillation therapy. Early signals of this possibility were that randomisation to a defibrillator was associated with an increased risk of rehospitalisation in AVID, and with a higher rate of new or worsening heart failure in MADIT II. Subsequent analyses of several ICD trials, including DINAMIT and MADIT II, demonstrated an association between ICD shocks (both appropriate and inappropriate) and ACM [25, 29, 30]. Evidence supporting a causative relationship arrived with the publication of the Multicenter Automatic Defibrillator Implantation Trial–Reduce Inappropriate Therapy (MADIT-RIT) study in 2012, in which randomisation to either of two novel ICD programming modes—both of which reduced appropriate and inappropriate ICD therapy—resulted in lower ACM than with randomisation to conventional ICD programming (which was most similar to the ICD programming used in the RCTs discussed above) [31•].

The question of who might benefit from receiving an ICD within 40 days of MI is currently being readdressed in the PROTECT-ICD trial (see Table 2). In the intervention arm of the trial, patients within 2–40 days of an MI will undergo an EP study, whereby those with inducible VT will receive an ICD, and those without shall not. Participants will alternatively be randomised to a control arm (standard care). In a sub-study of the trial, cardiac magnetic resonance (CMR) imaging will be performed, for correlation of CMR data with the presence of inducible VT at EP study and clinical outcomes. The primary endpoint of the trial is SCD or non-fatal arrhythmia, which may garner criticism given the issues discussed above.

**Table 2:** Selected ongoing research studies investigating the role of an ICD

Study name	Design	Population	Question to be answered	LVEF criteria	Primary outcome measure(s)	Intended enrollment	Estimated completion date	Location	ClinicalTrials.gov Identifier
PRE-DETERMINE: Biologic Markers and MRI SCD Cohort Study	Prospective cohort study	Patients with CAD or prior MI	Can biologic markers and ECG data (and for a subset, CMR data) advance SCD risk prediction	35–50%*	SCD or resuscitated VF	5764	2021	USA and Canada	NCT01114269
Cardiac Magnetic Resonance GUIDEd Management of Mild-moderate Left Ventricular Systolic Dysfunction (CMR-GUIDE)	RCT involving randomisation to either ICD or ILR	Patients with ventricular scar/fibrosis on CMR	Does scar/fibrosis on CMR identify patients with relatively preserved LVEF who will benefit from an ICD	36–50%	Composite of SCD + haemodynamically significant VA	1055	2022	Europe and Australia	NCT01918215
IA PRospective, randomised Comparison of subcuTaneOous and tRansvenous ImplANtable Cardioverter Defibrillator Therapy (PRAETORIAN)	RCT involving randomisation to transvenous ICD or subcutaneous ICD	Patients with indication for ICD (without indication for pacing)	Does a subcutaneous or transvenous ICD reduce major adverse events (ie inappropriate shocks, lead- or device-related complications)	Any	Number of participants with ICD-related adverse events	850	2019	USA and Europe	NCT01296022
MIBG Scintigraphy as a Tool for Selecting Patients Requiring Implantable Cardioverter Defibrillator (ICD) (MISTIC)	Prospective cohort study	Patients with indication for primary prevention ICD	Amongst ICD recipients, does evaluation of cardiac sympathetic innervation via MIBG scintigraphy identify those who will not require therapy for VAs	≤ 35%	MIBG uptake ratio (for identification of patients at very low risk of severe VAs)	330	2018	France	NCT01185756
International Study to Determine if AdreView Heart Function Scan Can be Used to Identify Patients With Mild or Moderate Heart Failure (HF) That Benefit From Implanted Medical Device (ADMIRE-ICD)	RCT involving randomisation to MIBG scintigraphy-guided allocation to an ICD or no ICD; or ICD as standard care	Patients with NYHA class II or III symptoms + indication for primary prevention ICD	Does an MIBG scintigraphy-guided strategy prevent ICD implantation in patients with a conventional indication but who will not benefit from receiving one	25–35%	ACM	2001	2021	North America and Europe	NCT02656329
Efficacy of Implantable Defibrillator Therapy After a Myocardial Infarction (REFINE-ICD)	RCT involving randomisation to ICD or no ICD	Patients with prior MI (> 2 months) + abnormal HRT and TWA on Holter monitor	Can non-invasive risk markers identify patients with relatively preserved LVEF who will benefit from an ICD	36–50%	ACM	1000	2021	North America, Europe and `Middle East	NCT00673842
Programmed Ventricular Stimulation to Risk Stratify for Early Cardioverter-Defibrillator (ICD) Implantation to Prevent Tachyarrhythmias Following Acute Myocardial Infarction (PROTECT-ICD)	RCT involving randomisation to EPS-guided allocation to ICD or no ICD; or standard care	2–40 days (inclusive) following an MI	Does an EPS-guided strategy identify patients who would benefit from receiving an ICD within 40 days of an MI	≤ 40%	SCD or non-fatal arrhythmia	1058	2023	USA, Europe and Australasia	NCT03588286

*30–35% if NYHA class I + no history of VAs

*HRT*, heart rate turbulence; *TWA*, T-wave alternans

### Patients Undergoing Coronary Artery Bypass Grafting

The Coronary Artery Bypass Grafting Patch (CABG-Patch) trial examined whether an ICD could reduce mortality in patients undergoing coronary artery bypass graft (CABG) surgery [6]. Eligible patients were already scheduled for CABG and required to have LVEF ≤ 35% and abnormalities on an SAECG. Nine hundred participants were randomised to peri-operative implantation of an epicardial ICD or standard care in a 1:1 ratio. During an average follow-up of 32 months, there were 101 deaths from any cause in the ICD group and 95 in the control group (HR 1.07, 95%CI 0.81–1.42). This was despite a borderline significant reduction in SCD with an ICD (HR 0.55, 95%CI 0.29–1.03; *p* = 0.06). Underlying this, SCD comprised just 29% of all deaths in the control group (compared to, for example, 49%, 50% and 51% in MADIT II, DINAMIT and IRIS respectively). Resultantly, it was more challenging for a defibrillator to significantly reduce SCD, and harder still for any such reduction to affect ACM. The explanation for this low rate of SCD is not certain. However, it was notable that the difference in the rates of SCD between the groups increased with duration of follow-up, with the Kaplan-Meier curves for SCD separating after ~ 6 months and diverging progressively over the follow-up period. A possible explanation is that revascularisation at the time of randomisation temporarily negated a potent substrate for arrhythmic death, since it has been estimated that 35–58% of SCD is preceded by myocardial ischaemia [32, 33]. By contrast, revascularisation was not universal in MADIT II, IRIS or DINAMIT, with a third of patients in DINAMIT receiving no revascularisation following their index MI. The implication is that, without a method to more precisely identify patients at high risk of SCD post-CABG, the rate of fatal VA in this population is too low to warrant a broad indication for a peri-operative defibrillator implantation. Because the rate of SCD increased as a function of time from revascularisation in CABG-Patch—presumably due to the development of graft failure—it would seem logical for a follow-up trial to have examined the potential benefit of an ICD in patients more remote from their index surgical procedure. However, a large proportion of this population was represented in the subsequent MADIT II trial, in which over half of patients had a previous CABG, thus diluting this impetus.

### Patients with Heart Failure and Reduced Ejection Fraction (HFrEF)

The Sudden Cardiac Death in Heart Failure Trial (SCD-HeFT) was the first RCT to examine whether an ICD would reduce the risk of death in a population with chronic heart failure and reduced ejection fraction (HFrEF) of either ischemic or non-ischemic aetiology (see Table 1 for trial characteristics) [15•]. The investigators also sought to determine whether the efficacy of an ICD was influenced by heart failure symptom severity. Two thousand five hundred twenty-one patients in NYHA functional classes II to III were randomised to placebo, amiodarone or an ICD. Over a median follow-up of 46 months, ICD treatment reduced ACM by 23% compared to placebo (HR 0.77, 97.5% CI 0.62–0.96, *p* = 0.007). The survival benefit conferred by an ICD did not differ according to ischemic or non-ischemic aetiology (*P* for interaction = 0.68), implying a survival advantage in patients with NICM. This benefit was maintained for patients in NYHA functional class II (HR 0.54, 97.5% CI 0.40–0.74, *p* < 0.001), but not for those in NYHA class III (*P* for interaction < 0.001). One potential explanation for this finding is that the competing risk of pump failure death in NYHA functional class III patients was too substantial for ICDs to reduce overall mortality. This possibility is supported by other studies in which patients with more advanced symptoms were more likely to die from pump failure than SCD [34–36]. However, the HR for SCD and other cardiovascular death (CVD) subtypes stratified by NYHA class were not published, and moreover, this finding was not replicated in MADIT II, in which there was no interaction between treatment effect and NYHA class [37].

The survival benefit of an ICD in relation to NYHA class was examined further in a recent patient-level meta-analysis of four primary prevention trials [38]. For the 1867 patients in NYHA class II, an ICD was associated with a reduction in ACM (HR 0.55, 95%CI 0.35–0.85). ACM was not significantly reduced for ICD-treated patients in NYHA class III (HR 0.76, 95%CI, 0.48–1.24); however, there were substantially fewer patients in this subgroup (*n* = 896), and the point estimate suggests a significant effect might have been proven had the group been larger. To add context to this issue, the distinction between NYHA functional classes is subjective, and individual patients within the same NYHA class may be at substantially different risk of sudden and non-sudden death according to other important characteristics, such as LVEF and the presence of severe comorbidity. Appropriately, international guidelines regarding primary prevention ICDs make no overriding distinction between recommendations for patients in NYHA functional class II or III (see Fig. 1). However, it is widely assumed that patients in NYHA class IV are at too high risk of pump failure death to benefit from an ICD. These patients were largely excluded from the above trials.

The Comparison of Medical Therapy, Pacing, and Defibrillation in Heart Failure (COMPANION) trial randomised 1520 patients with HFrEF to CMT alone or in combination with either a cardiac resynchronisation pacemaker (CRT-P) or cardiac resynchronisation defibrillator (CRT-D) [14]. Patients were in either NYHA functional classes III or IV, and both ICM and NICM patients were included. Across the trial population, assignment to CRT-D was associated with a 36% reduction in ACM when compared to CMT (HR 0.64, 95%CI 0.48–0.86, *p* = 0.003). This was driven in part by a 56% reduction in SCD (HR for CRT-D vs CMT 0.44, 95%CI 0.23–0.86, *p* = 0.02). It should be noted that these HRs incorporate the extra mortality benefit of randomisation to CRT in addition to that derived from a defibrillator. Specifically, amongst patients with NICM, CRT-D therapy led to a lower risk of ACM, as compared with CMT (HR 0.50, 95%CI 0.29–0.88). There was no interaction between ischemic/non-ischemic aetiology of HFrEF and the survival benefit of CRT-D over pharmacologic therapy.

### Patients with HFrEF and a Presumed Non-ischemic Aetiology: the Pre-DANISH Era

The first two primary prevention ICD trials (the Cardiomyopathy Trial (CAT) and Amiodarone Vs Implantable Cardioverter-Defibrillator (AMIOVIRT) trial) focussing on non-ischemic cardiomyopathy (NICM) were stopped early for futility, resulting in part from mortality rates that were far lower than anticipated [11, 12]. In CAT, for example, the rate of ACM after 12 months was just 5.6% in the control group, and although final rates of SCD were not reported, none occurred during the first 2 years. The Defibrillators in Non-Ischemic Cardiomyopathy Treatment Evaluation (DEFINITE) trial subsequently assigned 458 patients with NICM to medical therapy with or without a primary prevention ICD. Assignment to an ICD reduced SCD (HR 0.20, 95%CI 0.06–0.71, *p* = 0.006), but ACM was not significantly different between the groups (HR 0.65, *p* = 0.08) [13]. This was because the incidence of SCD was lower than anticipated, comprising 35% of all deaths in the control group. Over a mean follow-up of 29 months, there were only 68 deaths from any cause. Results from the SCD-HeFT and COMPANION trials involving subgroups with NICM are discussed above.

The CAT, AMIOVIRT, DEFINITE and SCD-HeFT and COMPANION trials were subsequently combined in a meta-analysis, which was until recently the most robust evidence available regarding the efficacy of ICDs in NICM [39]. Pooled analysis indicated a reduction in ACM amongst patients randomised to a defibrillator (either ICD or CRT-D) when compared to medical therapy (HR 0.69, 95%CI 0.55–0.87, *p* = 0.002). It is important to note that the data utilised in this meta-analysis from COMPANION were a comparison of CRT-D and medical therapy, since the effect of CRT-D versus CRT-P was not available at the time.^3^ Since CRT reduces both SCD and other forms of death in patients with heart failure [40], the perceived efficacy of a defibrillator in this analysis may have been overestimated by the concomitant benefit of CRT.

Despite the lack of an RCT showing outright benefit of an ICD in patients with NICM, current ESC guideline recommendations for primary prevention ICDs in patients with HFrEF (published 2015 and based on MADIT II, SCD-HeFT, DEFINITE and the meta-analysis discussed above) vary little according to ischemic/non-ischemic aetiology (see Fig. 1). The ACC/AHA/HRS guidelines were published in 2017, subsequent to the results of the DANISH trial, which is discussed next.

### Patients with HFrEF and a Presumed Non-ischemic Aetiology: the DANISH Trial

The largest and most recent primary prevention ICD trial in patients with NICM was the Danish Study to Assess the Efficacy of ICDs in Patients with Non-ischemic Systolic Heart Failure on Mortality (DANISH) [16•]. DANISH randomised 1116 patients to optimal medical therapy with or without a defibrillator in a 1:1 allocation. After a median follow-up period of 5.6 years, ACM did not differ significantly between the defibrillator and control groups, in which there were 120 and 131 deaths respectively (HR, 0.87, *p* = 0.28), despite the fact that an ICD conferred a 50% reduction in the risk of SCD (HR 0.50, 95%CI 0.31–0.82, *p* = 0.005).

What can we learn from DANISH? In this contemporary trial, SCD constituted 35% of overall mortality in the control group, matching the proportion found in DEFINITE. However, whilst the annual mortality rate in DEFINITE was low at 7%, it was just 5% in DANISH. Resultantly, there were only 70 sudden deaths overall, with the rate of SCD in the control group less than 2% per year. This means the absolute reduction in SCD was just 3.9% during a follow-up of more than 5 years. More broadly, CVD comprised only 73% of ACM amongst patients in the control group, a relatively small proportion when set against older ICD trials, and heart failure trials more generally (82% in MADIT II, for instance).

Underlying these low mortality rates, the use of neurohormonal antagonist drugs with prognostic benefit in heart failure was high in DANISH, with angiotensin-converting enzyme (ACE) inhibitors or angiotensin II receptor blockers (ARBs), beta-blockers and mineralocorticoid receptor antagonists (MRAs) prescribed to 97%, 92% and 58% of patients respectively. In parallel, 58% of patients in both treatment arms were receiving cardiac resynchronisation therapy (CRT). In patients with heart failure, these pharmacologic therapies and CRT each additively reduce the risk of both SCD and pump failure death, and indeed of CVD more generally. As these treatments are increasingly more prevalent, rates of both sudden and cardiovascular death are falling across RCTs enrolling patients with heart failure [41, 42]. Resultantly, in an optimally treated trial population, even a large reduction in SCD may fail to significantly impact overall mortality if SCD is a relatively small proportion of CVD, and CVD is in turn a relatively small proportion of ACM. For trialists seeking to show that an ICD—a treatment which reduces SCD but not other modes of death—confers an overall survival benefit, this increases the challenge.

In this context, recent analysis of patients receiving the most optimal medical therapy in COMPANION yields a salient observation [43**•**]. Across the entire trial cohort, the reductions in ACM with CRT-D and CRT-P when each was compared to CMT were 36% and 24% respectively (CRT-D vs CMT: HR 0.64, 95%CI 0.48–0.86; CRT-P vs CMT: HR 0.76, 95% CI 0.57–1.01). However, amongst patients receiving two neurohormonal antagonists (ACE-inhibitor or ARB, plus beta-blocker; ~ 61% of patients in COMPANION), the difference in treatment efficacy between CRT-D and CRT-P was abolished (CRT-D vs CMT: HR 0.56, 95%CI 0.37–0.85; CRT-P vs CMT: HR 0.57, 95%CI 0.37–0.85). A similar trend was evident in the much smaller subgroup of patients who were additionally receiving an MRA. The implication is that in the best-treated patients, a defibrillator confers no incremental benefit on top of CRT plus optimal medical therapy. This is supported by a recent meta-analysis of COMPANION and DANISH, in which there was no significant difference in ACM between patients randomised to CRT-P or CRT-D [44].

A declining rate of CVD associated with optimal medical therapy also lends greater weight to the contribution of non-cardiovascular death to ACM. Since comorbidity associates with older age, a proportionately greater incidence of non-cardiovascular death is liable to emerge in trials with longer follow-up and lower CVD, such as DANISH. In keeping with this, there was a significant interaction between age and the treatment effect of an ICD in DANISH, with benefit suggested for patients in the younger two tertiles, but not in the older tertile: age < 59 years (HR 0.51, 95%CI 0.29–0.92), age ≥ 59 to < 68 years (HR 0.75, 95%CI 0.48–1.16) and ≥ 68 years (HR 1.19, 95%CI 1.19–1.73) (*P* for interaction = 0.009). Moreover, the investigators reported that the proportional hazard assumption for ACM was violated (*p* = 0.054 when tested with Schoenfeld residuals), implying that the benefit derived from treatment with an ICD varied as the trial progressed. Reflecting this, the Kaplan-Meier curves diverged during the first 5 years, before converging and eventually crossing after ~ 7.5 years. In totality, this suggests that ICDs reduce overall mortality in younger patients before the development of worsening heart failure and/or non-cardiovascular comorbidity, leading to an increasing risk of non-sudden death. A recent pre-specified subgroup analysis by the DANISH investigators further examined the interaction between age and ICD benefit [45]. This demonstrated that with an ICD, each year of younger age was associated with a 3.0% (95%CI, 0.03–6.0; *p* = 0.03) lower relative risk of ACM, with the point estimate crossing unity at ~ 70 years of age. When dichotomised, patients ≤ 70 years of age had significantly better survival with an ICD (HR, 0.70; 95%CI, 0.51–0.96; *p* = 0.03) whereas those > 70 years old did not (HR 1.05; *p* = 0.84). The rates of sudden and non-sudden death in the control group were 1.8 and 2.7 events per 100 patient-years respectively in those aged ≤ 70 years. In contrast, they were 1.6 and 5.4 events per 100 patient-years respectively for those aged > 70 years.

## Where Do We Go From Here?

The DANISH trial reminds us that the evolution of pharmacologic therapy and CRT has altered the landscape in which trialists seek to prove a survival benefit with ICD therapy. Only where there is a sufficiently high risk of arrhythmic death, and concomitantly low risk of all other forms of death, can the capacity for ICDs to reduce SCD translate into an observable reduction in ACM. But the majority of patients with heart failure and/or LVSD no longer die from arrhythmic causes, and CVD has also proportionally declined, resulting in non-cardiovascular mortality playing an increasingly prominent role. Insights from DANISH affirm that increasing age and the development of comorbidity identify patients at higher risk of non-sudden death i.e. those less likely to benefit from a defibrillator. As time moves on, so competing risks of death emerge. In this regard, the much shorter follow-up periods employed in earlier ICD trials are notable: in MADIT II and COMPANION for instance, the durations of follow-up were just 20 and ~ 15 months respectively. Would the benefit of an ICD in these RCTs have been so great had trial follow-up been longer?

This question arises within the broader issue of whether evidence from historic ICD trials remains enduringly robust in the modern era. Across the key trials of both primary and secondary prevention therapy (amongst the latter group of which it should be noted that not all patients had LVSD), prescription rates of ACE-inhibitors, ARBs, beta-blockers and MRAs were either sub-optimal by contemporary standards or not recorded (in which scenario utilisation was likely low). Moreover, sacubitril-valsartan was not yet available and few patients would have received CRT, which was still an investigational device during the enrolment periods of most of these trials. If the RCTs upon which the guidelines are based were repeated today, how might this range of prognostic therapies, appropriately applied, affect the result? Randomised data regarding the benefit of catheter ablation for secondary prevention of ventricular arrhythmias are lacking; however, this treatment too would now be available for the control group if the secondary prevention ICD trials were re-run. An updated trial of primary prevention ICDs in patients with ICM will surely be performed at some point. Will anyone be courageous enough to perform a similar trial of secondary prevention ICDs?

A pressing challenge is to better identify patient subgroups likely to benefit from an ICD beyond relatively blunt risk stratifiers such as LVEF, NYHA classification and ischemic aetiology of heart failure. A vast array of parameters has been evaluated without overwhelming success, including laboratory indices and biomarkers, electrocardiographic and functional variables, novel echocardiographic techniques such as global longitudinal strain, tissue characterisation by CMR and special imaging modalities such as Iodine-123 *meta*-iodobenzylguanidine (_123_I-*m*IBG). A selection of ongoing research studies is presented in Table 2. These largely focus on refining patient selection within populations with an existing indication for a defibrillator, or identifying patients who might benefit amongst populations without an existing indication e.g. primary prevention ICDs in patients with LVEF > 35%. In the former category, the challenge will be to sub-select patients with the lowest competing risk of non-sudden mortality amongst those with established high risk of SCD. In the latter category, the competing risk of pump failure death will certainly be lower than in patients with LVEF ≤ 35%, but will their risk of arrhythmic SCD be sufficiently high? In a recent study of 912 unselected ‘sudden’ deaths in the United States, rigorous application of World Health Organisation diagnostic criteria for SCD by investigators resulted in the reclassification of >40% of deaths as non-sudden. [46] Less than half of presumed SCDs were adjudicated as being of arrhythmic aetiology. This suggests that the capacity of an ICD to reduce sudden deaths amongst populations without an existing indication for a defibrillator may be lower than might otherwise be assumed.

For completeness, it should also be noted that, although relatively rare, proarrhythmic gene defects (e.g. Lamin A/C) and other inherited primary arrhythmia syndromes (e.g. the Brugada and Long or Short QT Syndromes) may occasionally identify patients at sufficiently high risk of SCD to warrant an ICD. Similarly, guideline recommendations exist [19, 20] for ICD use in a range of structural heart diseases (e.g. certain congenital heart defects and hypertrophic, restrictive, or arrhythmogenic right ventricular, cardiomyopathies), systemic diseases with cardiac involvement (e.g. sarcoidosis) and several other special populations (such as certain patients with a ventricular assist device or awaiting—or in receipt of—a cardiac transplant). There is a paucity of robust evidence for such niche indications, which are themselves beyond the scope of this review.

A final question is the extent to which further advances in the ICD itself might be important. The development of transvenous systems, anti-tachycardia pacing, biphasic shock generators, improved battery technology (limiting the need for generator replacements), telemetry capability, and most recently the emergence of superior programming configurations, have refined the risk-benefit ratio intrinsic to the devices themselves (as opposed to that conferred by the characteristics of the recipient). Amongst patients in the AVID trial who died suddenly despite an ICD, there was the suggestion that a significant proportion of deaths was attributable to the failure of the defibrillator to terminate a VA [47]. By contrast, in the modern era ICDs abort ≥ 95% of malignant VAs [48]. Similarly, considering the supposition that treatment with an ICD might have directly increased non-sudden cardiac mortality in earlier ICD trials, such a trend was not detectable in DANISH, which thus far is the only primary prevention trial to have implemented ‘MADIT-RIT programming’, and consequently reported a comparatively low number of inappropriate shocks. It is unclear how much benefit can be wrought from further technical improvements in the functionality of an ICD, but it seems likely that most of the gains have already been made. The place of alternative ICD delivery systems—namely subcutaneous devices and non-implanted, wearable devices—remains to be fully elucidated and detailed discussion of them is beyond the remit of this review.

## Conclusion

Which patients stand to benefit from an ICD? At first glance, this would appear to be patients with a high risk of arrhythmic SCD and relatively low risk of other modes of death. This ostensibly leads to the assumption that ICDs must reduce mortality for populations in which these characteristics are observed. However, as suggested in DINAMIT and IRIS, patients who require defibrillator shocks to abort malignant VAs may be at higher risk of other modes of death than apparently similar patients who do not. Therefore, a more robust answer is: those patients with a sufficiently high risk of arrhythmic SCD and sufficiently low risk of all other modes of death *even when treated with a defibrillator*. The determination of whether an ICD reduces mortality is thus impossible to make outside of a randomised trial. Hazard ratios for ACM in the major primary and secondary prevention RCTs reflect broad populations fitting this description. However, scrutiny of the evidence affirms that decisions regarding an ICD should be tailored to individual risk, regardless of whether that person meets the wider inclusion criteria of a positive trial. Consideration should be made, for example, of age, aetiology of heart failure and/or presence of CAD, history of syncope, NYHA functional class, severity of comorbidity, tolerated pharmacologic therapy and the presence of—or suitability for—CRT. To differing degrees, this premise holds true regardless of the indication for a defibrillator. The complexity of this situation has been compounded by the revolution in prognostic therapies for LVSD with or without heart failure over the past three decades, which means that the absolute validity of historical trial evidence is open to question. It is increasingly obvious that new RCTs are necessary, and the possible variations upon these are extensive [49]. What remains to be seen is how far trial investigators will dare to go in re-challenging the evidence base.

## Footnotes

1 Assignment to propafenone was terminated early due to increased mortality after 58 patients had been randomised to this arm, with results thus reported for the remaining 288 patients in the defibrillator, amiodarone and metoprolol arms.

2 It was due to this realisation that, midway through the CIDS trial, the Steering Committee revised the primary outcome measure from arrhythmic death to ACM

3 Recently, a comparison of the CRT-D and CRT-P arms from COMPANION was published [43•]: the HR for ACM was 0.84 (95%CI 0.65–1.09), for CVD it was 0.73 (95%CI 0.55–0.98) and for SCD it was 0.37 (95%CI 0.21–0.65).
